# The World of Algae Reveals a Broad Variety of Cryptochrome Properties and Functions

**DOI:** 10.3389/fpls.2021.766509

**Published:** 2021-11-01

**Authors:** Jan Petersen, Anxhela Rredhi, Julie Szyttenholm, Sabine Oldemeyer, Tilman Kottke, Maria Mittag

**Affiliations:** ^1^Matthias Schleiden Institute of Genetics, Bioinformatics and Molecular Botany, Friedrich Schiller University, Jena, Germany; ^2^Experimental Molecular Biophysics, Department of Physics, Freie Universität Berlin, Berlin, Germany; ^3^Department of Chemistry, Bielefeld University, Bielefeld, Germany; ^4^Biophysical Chemistry and Diagnostics, Medical School OWL, Bielefeld University, Bielefeld, Germany

**Keywords:** blue-light receptor, *Chlamydomonas*, flavin, *Ostreococcus*, *Phaeodactylum*, photoreceptor, photosynthetic microorganisms

## Abstract

Algae are photosynthetic eukaryotic (micro-)organisms, lacking roots, leaves, and other organs that are typical for land plants. They live in freshwater, marine, or terrestrial habitats. Together with the cyanobacteria they contribute to about half of global carbon fixation. As primary producers, they are at the basis of many food webs and they are involved in biogeochemical processes. Algae are evolutionarily distinct and are derived either by primary (e.g., green and red algae) or secondary endosymbiosis (e.g., diatoms, dinoflagellates, and brown algae). Light is a key abiotic factor needed to maintain the fitness of algae as it delivers energy for photosynthesis, regulates algal cell- and life cycles, and entrains their biological clocks. However, excess light can also be harmful, especially in the ultraviolet range. Among the variety of receptors perceiving light information, the cryptochromes originally evolved as UV-A and blue-light receptors and have been found in all studied algal genomes so far. Yet, the classification, biophysical properties, wavelength range of absorbance, and biological functions of cryptochromes are remarkably diverse among algal species, especially when compared to cryptochromes from land plants or animals.

## Introduction

### What Are Algae?

Algae are photosynthetic eukaryotes defined primarily by their lack of roots, leaves, and the stem that are typical for higher plants (Parker et al., 2008). They are divided into microscopically small microalgae that are a major part of phytoplankton and macroalgae, including seaweeds. Algae are found anywhere from soil to lakes, rivers and oceans and they are crucial to food webs (Steele, 1974). Algal activities can even influence biogeochemical processes as observed with the Greenland ice sheet (McCutcheon et al., 2021). Together with cyanobacteria, aquatic algae are responsible for about 50% of global carbon fixation (Field et al., 1998) and thus are of high ecological relevance. Evolutionarily, algae can be divided into two major groups, with one group derived from primary endosymbiosis and the other one from secondary endosymbiosis (Keeling et al., 2005). For primary endosymbionts, a unicellular eukaryotic cell engulfed a cyanobacterium to become a chloroplast. In the case of secondary endosymbionts, a unicellular eukaryotic cell engulfed either a green alga (secondary green) or a red alga (secondary red). Recently, it was shown that tertiary and possibly even higher-order endosymbiotic events occurred (Sibbald and Archibald, 2020), but these will not be part of this review. Primary endosymbionts (named Archaeplastida) include glaucophytes (algae with cyanelle plastids), rhodophytes (red algae), chlorophytes (green algae), and all land plants together with ferns and mosses (Bowman et al., 2007; Adl et al., 2012). Green algae and land plants are also grouped as Chloroplastida. All these groups possess plastids surrounded by two envelope membranes. In the lineages with secondary endosymbionts, plastids are encircled by either four (e.g., diatoms, brown algae, haptophytes, and cryptophytes) or three membranes (euglenophytes, dinoflagellates; Gentil et al., 2017). Some of these lineages still contain the nucleus of the engulfed green or red alga, but in a strongly reduced form, called nucleomorph (e.g., in cryptophytes). Others such as euglenophytes, diatoms, dinoflagellates, or brown algae have completely lost this nucleus (Gentil et al., 2017). In this review, we focus solely on algal groups with cryptochromes that have been so far phylogenetically described based on existing genomes or have been characterized by their photoreceptors.

### The Influence of Light on Algal Life and the Diversity of Algal Photoreceptors

**Figure 1:**
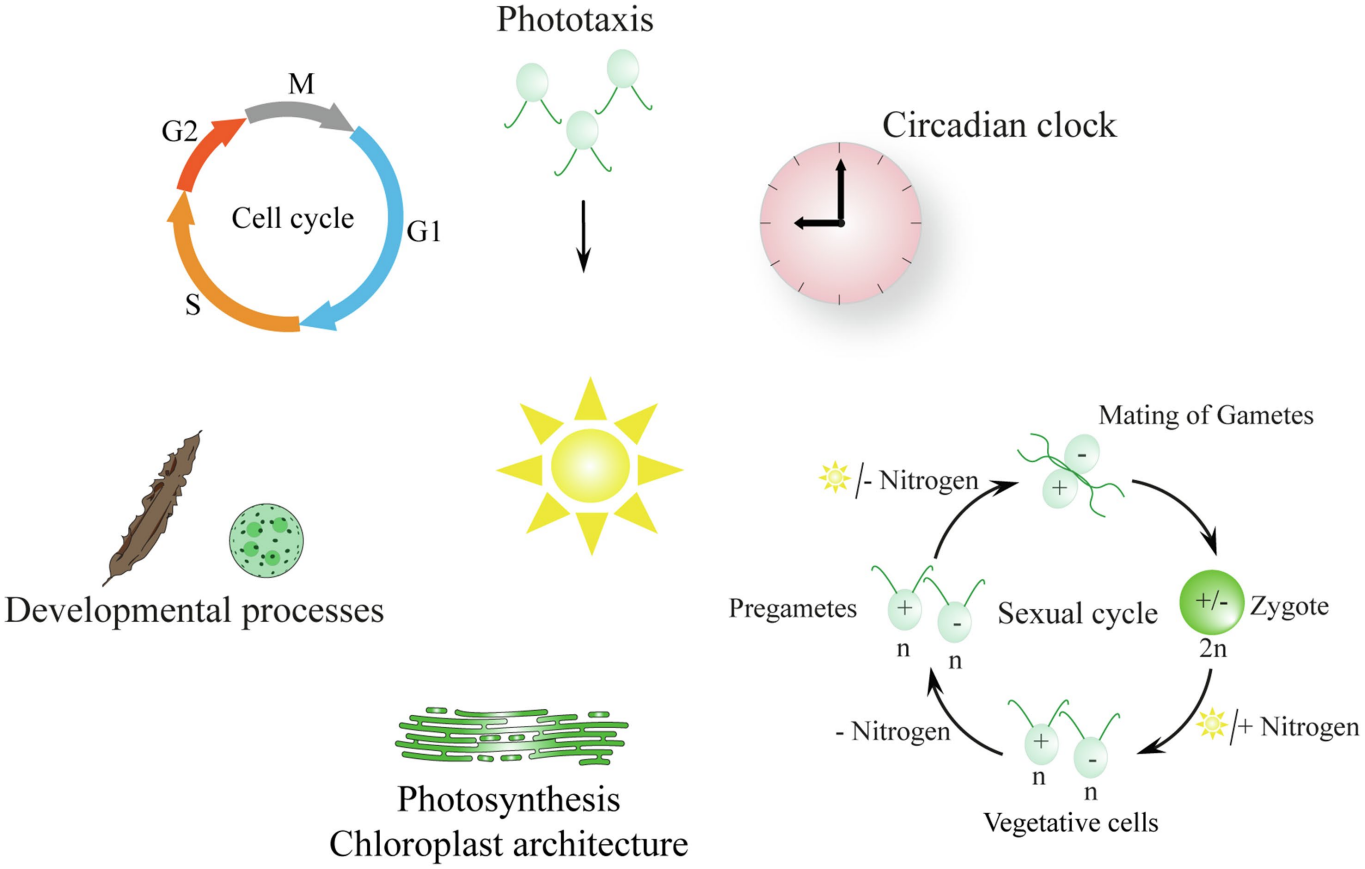
Biological processes in algae that are regulated by light (reviewed in Hegemann, 2008; Kianianmomeni and Hallmann, 2014; Kottke et al., 2017; Sasso et al., 2018). The description of the symbolized processes begins at the top of the scheme and moves in a clockwise manner: Photoorientation of flagellate algae: tactic movement to the light is shown; phobic movement occurs with strong light (not shown). ǀ The algal circadian clock is entrained by light–dark cycles. ǀ The sexual cycle of *Chlamydomonas*: vegetative cells turn into pregametes in the dark without a nitrogen source and then become gametes in the light in the absence of a nitrogen source; gametes fuse and a resilient zygote is formed that needs a nitrogen source and light to undergo meiosis. ǀ Light is needed for photosynthesis and influences the chloroplast architecture; thylakoid membranes are shown exemplarily (Rredhi et al., 2021) ǀ Light influences developmental processes in the multicellular algae *Saccharina* and *Volvox* (Kianianmomeni and Hallmann, 2015; Yang et al., 2020). ǀ The cell cycle is symbolized; algal cells are synchronized by light–dark cycles; M, mitosis; G1, gap1; S, synthesis; G2, gap2 (Coesel et al., 2009; Cross and Umen, 2015).

Light is an important source of energy and information for algal life on our planet (Figure 1). As for all photosynthetic organisms, algae transform the radiation energy of the sunlight into chemical energy (Eberhard et al., 2008). They perceive light *via* pigments or specialized photoreceptors. Light regulates their photosynthesis and balances their photosynthetic apparatus, controls their behavior (photoorientation), entrains their circadian clocks, and influences their cell and sexual cycles as well as developmental processes (reviewed in Hegemann, 2008; Kianianmomeni and Hallmann, 2014; Kottke et al., 2017; Rredhi et al., 2021).

Cyanobacteria, also known as prokaryotic blue-green algae, and eukaryotic algae have an amazing repertoire of different photoreceptors compared to land plants or animals. For many members, we still do not know their detailed functions and/or mechanisms (Kianianmomeni and Hallmann, 2014; Greiner et al., 2017; Jaubert et al., 2017; Kottke et al., 2017; Kroth et al., 2017; König et al., 2017b; Rockwell and Lagarias, 2020). In addition to the classically known photoreceptors from land plants which absorb in the red/far red (phytochrome) and blue region of the visible spectrum (cryptochrome, phototropin, Zeitlupe) as well as in the UV-B (UVR8; Kim et al., 2007; Fankhauser and Christie, 2015; Ahmad, 2016; Wang and Lin, 2020; Podolec et al., 2021), algae bear several novel types of light sensors. Moreover, cyanobacteria and algae also possess a larger variety of the above-mentioned “classical” photoreceptors with new properties and biological functions. For example, cyanobacteriochromes, which are phytochromes in cyanobacteria, absorb strongly shifted from the well known red/far-red range to shorter wavelengths of visible light (Rockwell et al., 2012). Moreover, new types of cryptochromes are present in algae with novel biophysical properties such as red-light absorption and will be detailed in the chapters below.

Numerous photoreceptors are unique to algae. For example, aureochrome photoreceptors are only present in a single group of algae, the photosynthetic stramenopiles, including Xanthophyceae (yellow-green algae), diatoms, and brown algae (Kroth et al., 2017). These aureochrome photoreceptors contain a light-oxygen-voltage (LOV) domain for light reception and a basic region leucine zipper (bZIP) domain for DNA binding and act as light-driven transcription factors. In the green unicellular model alga *Chlamydomonas reinhardtii* (Cr), even 18 different types of photoreceptors have been reported, including two channelrhodopsins that are fundamental to its photoorientation and several histidine kinase rhodopsins (HKRs) whose functions are largely unknown (Greiner et al., 2017; Luck and Hegemann, 2017). HKRs contain a His-kinase-, response regulator-, and a rhodopsin domain. Some of the HKRs even have guanylyl cyclase activity that has been recently used for application as an optogenetic tool (Tian et al., 2021). The field of optogenetics is self-evolving since knowledge on the exceptional properties of Cr channelrhodopsins became available (Hegemann and Nagel, 2013).

Further types of (possibly) novel light sensors were recently discovered in phytoplankton in the open ocean (Coesel et al., 2021). The genomes of these marine microalgae encode light-sensing proteins with new combinations of known domain structures or even fusions of different types of photoreceptors. For example, LOV domains were found within one protein together with different DNA-binding domains such as a homeobox or a heat shock factor domain or with signal-transduction motifs (e.g., an EF-hand; Coesel et al., 2021). A new type of dual cryptochrome that is fused to another photoreceptor was also found in these genomes (Makita et al., 2021) and is included in this review.

It should also be stated that the functions of photoreceptors in algae may differ from those in higher plants. For example, Cr phototropin influences the sexual cycle (Huang and Beck, 2003), the development of the eyespot, a primitive visual system of the alga (Trippens et al., 2012), and mediates the feedback regulation of photosynthesis in the green alga (Petroutsos et al., 2016).

Blue-light-activated adenyl cyclases represent another type of light sensors in algae. They mediate photoavoidance in the secondary green alga *Euglena gracilis* (Iseki et al., 2002) and contain blue-light sensor using flavin adenine dinucleotide (BLUF) domains (Ito et al., 2010). In the green alga Cr as well as in animal spermatozoa, the BLUF domain is directly associated with dynein and involved in ciliary motility (Kutomi et al., 2021).

## Variety of Algal Cryptochromes

In this review, we focus on the variety of cryptochromes found in algae. Cryptochromes are flavoproteins that were first described as blue-light receptors in plants and animals (Chaves et al., 2011). They are derived from the blue-light-dependent DNA repair enzymes called photolyases that can be found in pro- and eukaryotes (Ahmad and Cashmore, 1993; Sancar, 2003; Chaves et al., 2011). As primary light sensors, cryptochromes and photolyases carry a non-covalently bound flavin adenine dinucleotide (FAD) molecule within their photolyase homology domain (PHR). Additionally, they may bind an antenna chromophore like 5,10-methenyltetrahydrofolate (MTHF) or 8-hydroxy-7,8-didemethyl-5-deazaflavin (8-HDF). The antenna chromophore harvests additional light and transfers the energy to the FAD (Hoang et al., 2008). Photoexcitation of the FAD may lead to a change of its redox state that can be either oxidized (FAD_ox_), semireduced as an anion radical (FAD•^−^), semireduced as a neutral radical (FADH•) or fully reduced (FADH^−^; Chaves et al., 2011). Conformational changes are promoted within the cryptochrome protein structure depending on the FAD redox state, resulting in different signaling properties. Most cryptochromes possess a C-terminal extension (CCT) whose structure is rather undefined and only shows poor conservation. The CCT varies in length depending on the cryptochrome. Nevertheless, the CCT is of great importance for cryptochrome function and downstream signaling (Chaves et al., 2011). The first cryptochromes were analyzed as blue-light receptors in the land plant *Arabidopsis thaliana* (At CRY1 and At CRY2; Ahmad and Cashmore, 1993: Chaves et al., 2011) and in the fly *Drosophila melanogaster* (dCRY) as an animal-type I CRY (Emery et al., 1998). Later, it was found that the cryptochromes from mice or humans group to animal type II CRYs. They do not act as light sensors but instead have a function in the central oscillator of the circadian clock (Okamura et al., 1999; Todo, 1999; Chaves et al., 2011).

### Cryptochrome Categories in Algae

**Figure 2:**
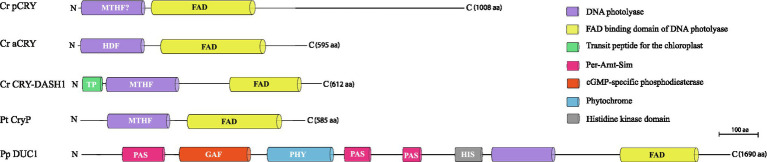
Cryptochrome categories in algae (Juhas et al., 2014; Kottke et al., 2017; Makita et al., 2021; Rredhi et al., 2021). A representative member of each of the five known categories is shown, starting with the classical plant cryptochrome of the green flagellate alga *Chlamydomonas reinhardtii* (Cr pCRY) and its long C-terminal extension. It is followed by the Cr animal-like CRY of the cryptochrome/photolyase family (Cr aCRY), the Cr CRY-DASH1 with its chloroplast transit peptide, the plant-like CryP of the diatom *Phaeodactylum tricornutum* and the dual cryptochrome DUC1 from the green picoalga *Pycnococcus provasolii*, a fusion of a phytochrome and a cryptochrome. Domains are indicated, including the names of the chromophores (FAD) and antenna chromophores (MTHF, 8-HDF); FAD, flavin adenine dinucleotide; MTHF, 5,10-methenyltetrahydrofolate; 8-HDF, 8-hydroxy-7,8-didemethyl-5-deazaflavin.

**Figure 3:**
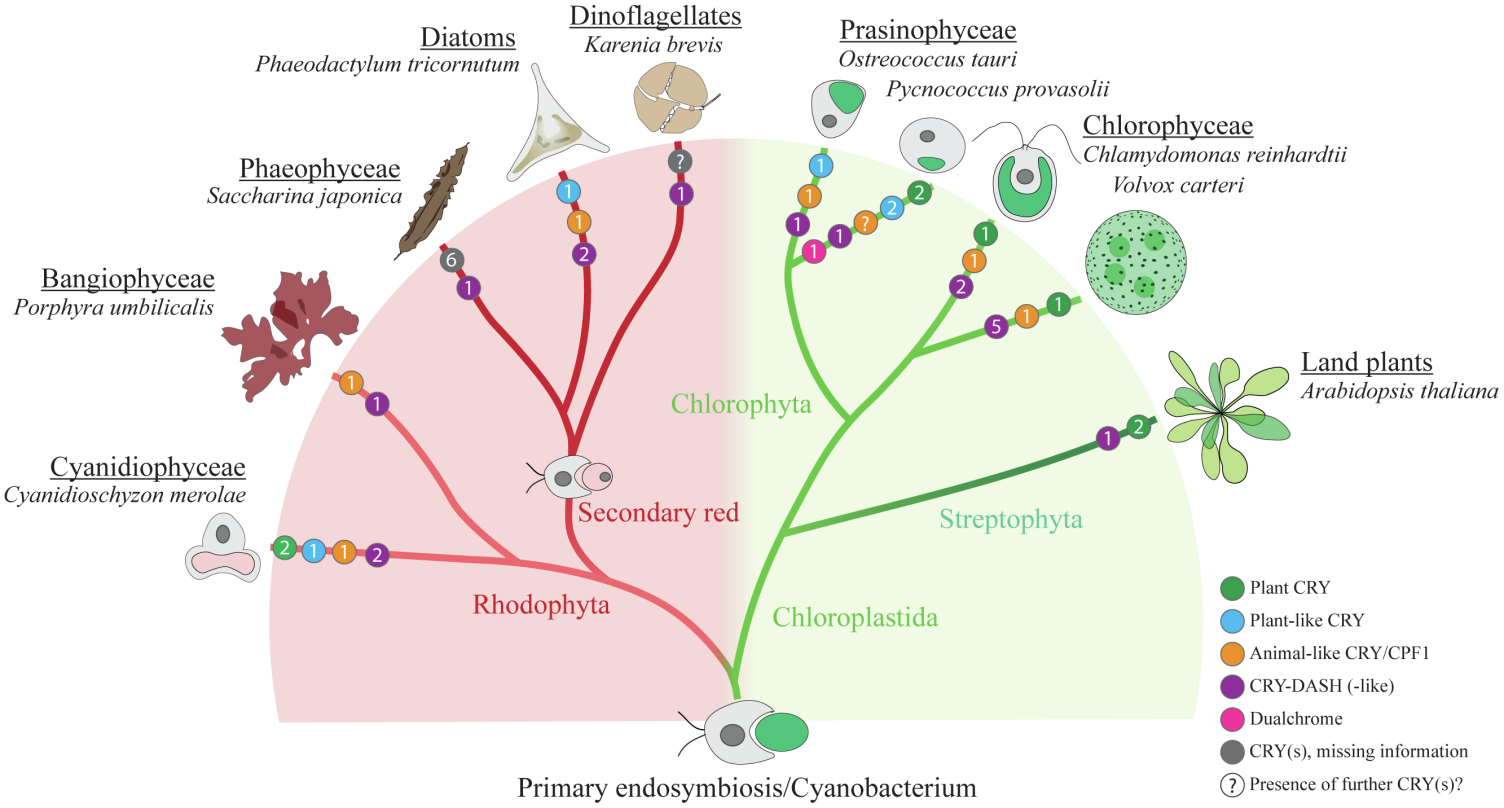
Cryptochrome distribution within selected algal species. Simplified schematic cladogram of cryptochrome (CRY) distribution within selected algal species in comparison with the land plant *Arabidopsis thaliana*. Colored circles indicate the numbers (presented in each circle) and representatives of different types of CRYs within the presented species (green, plant CRY; blue, plant-like CRY; orange, animal-like CRY/CPF1; purple, CRY-DASH and CRY-DASH-like; magenta, dualchrome; gray, CRY(s) that are currently not assigned to the former groups; “?,” indicating that there may be further potential CRY(s) in this organism that have not been identified so far). Information for the presented data was taken from the following references: *Cyanidioschyzon merolae* (Cm; Brawley et al., 2017), *Porphyra umbilicalis* (Pum; Brawley et al., 2017), *Saccharina japonica* (Sj; Deng et al., 2012; Yang et al., 2020), *Phaeodactylum tricornutum* (Pt; Juhas et al., 2014; Oliveri et al., 2014; Fortunato et al., 2015), *Karenia brevis* (Kb; Brunelle et al., 2007), *Ostreococcus tauri* (Ot; Heijde et al., 2010; Kottke et al., 2017), *Pycnococcus provasolii* (Pp; Makita et al., 2021), *Chlamydomonas reinhardtii* (Cr; Beel et al., 2012; Kottke et al., 2017), *Volvox carteri* (Vc; Kottke et al., 2017) and *Arabidopsis thaliana* (At; Chaves et al., 2011). Please note that three putative CRY-DASHs are predicted for Cm in the analysis of Asimgil and Kavakli (2012). For the genus *Karenia*, three putative CRY/photolyase members are predicted (Shikata et al., 2019). The number of cryptochrome/photolyase members from an additional dinoflagellate and from three stramenopile radiophytes, which all form noxious red tides (Shikata et al., 2019), has been omitted from the figure for clarity. As there is no data available of CRYs from the secondary green line, this line was omitted. The cladogram was modified after Handrich et al. (2017).

To date, four different classes of cryptochromes are known in algae based on phylogenetic analyses and further characterizations. Moreover, a fifth class was recently described consisting of a fusion of a cryptochrome with another photoreceptor. The following section will present details on the five different classes:

1. The classical plant cryptochromes (pCRYs) present in land plants (At CRY1 and At CRY2) are found in some but not in all algae. Plant CRYs, like all cryptochromes, share the conserved PHR at the N-terminus binding FAD (Chaves et al., 2011), and contain a comparatively long CCT (Reisdorph and Small, 2004; Müller et al., 2017). Cr pCRY has the longest known extension with about 500 amino acids (Figure 2). Plant CRYs may also bind MTHF as antenna chromophore as derived from their homology to CPD (cyclobutane pyrimidine dimer) photolyases (Chaves et al., 2011; Beel et al., 2012). There is no indication that pCRYs still have photolyase activity (Lin and Shalitin, 2003). Plant CRYs are found in many investigated Chloroplastida (Figure 3) but seem to be absent in the green picoalga *Ostreococcus tauri* (Ot), in the red macroalga *Porphyra umbilicalis* (Pum) or in the diatom *Phaeodactylum tricornutum* (Pt; Figure 3).
2. The cryptochrome–photolyase family (CPF1) of animal-like cryptochromes. For a long time, it was thought that all cryptochromes have lost photolyase activity. However, it was recently discovered that some algal animal-like CRYs from Pt, Ot, and Cr have maintained photolyase activity (Coesel et al., 2009; Heijde et al., 2010; Franz et al., 2018). These animal-like cryptochromes group by their phylogeny closely to the cryptochrome (6-4)–photolyase family (CPF1) and are thus phylogenetically located in between the animal-type I and -type II cryptochromes from animals (Beel et al., 2012). They exert further biological functions including light perception and circadian clock control as well as the control of the algal Cr life cycle (Coesel et al., 2009; Heijde et al., 2010; Beel et al., 2012; Zou et al., 2017). Cr aCRY binds 8-HDF as antenna chromophore (Franz et al., 2018; Figure 2). The CCTs of the animal-like CRYs are usually shorter as compared to the pCRYs. Notably, CPF animal-like CRYs are widely distributed in algae but are missing in land plants (Figure 3).
3. The CRY-DASH (*Drosophila*, *Arabidopsis*, *Synechocystis*, *Homo*) cryptochromes. CRY-DASH (-like) proteins are found in many organisms from bacteria to vertebrates (Brudler et al., 2003; Kiontke et al., 2020). They group closely to CPD photolyases (Beel et al., 2012; Fortunato et al., 2015). Bacterial, plant, and fungal CRY-DASHs can repair CPD in single-stranded and even in double-stranded DNA, as reported for some members of the mucoromycotina fungi (Selby and Sancar, 2006; Pokorny et al., 2008; Tagua et al., 2015; Navarro et al., 2020). Studies on repair activities by members of the cryptochrome/photolyase family in algal systems are rare. It was found that two out of three putative CRY-DASHs of the red picoalga Cm repair CPD lesions, but only in single-stranded and not in double-stranded DNA (Asimgil and Kavakli, 2012). Interestingly, CRY-DASH proteins can be located in organelles. The land plant At CRY3 was found in the chloroplast and in mitochondria (Kleine et al., 2003), while the algal Cr CRY-DASH1 protein was only found in the chloroplast (Rredhi et al., 2021; Figure 2). Recently, algal CRY-DASHs were shown to be involved in further biological functions beside DNA repair, supporting their additional or alternative role as photoreceptors (Yang et al., 2020; Rredhi et al., 2021). CRY-DASH proteins were found in all selected algal systems (Figure 3), including an Antarctic *Chlamydomonas* sp. ICE-L in which its highest transcript expression level is at 5°C and a salinity of 32% (Zhang et al., 2020).
4. The plant-like CryP, found in algae and metazoans. Instead of a classical pCRY, the genome of Pt encodes a plant-like CRY, named CryP (Juhas et al., 2014; König et al., 2017b) that was later identified in metazoan genomes (Oliveri et al., 2014). CryP binds FAD and MTHF as chromophores and has a CCT of about 70 residues (Figure 2). As in CRY-DASH proteins, the FAD in CryP is present in the neutral radical state after isolation as opposed to the oxidized state found in pCRYs (see below; Juhas et al., 2014). A plant-like CRY is also present in the picoalga Ot that lacks a classical pCRY. Interestingly, the red picoalga *Cyanidioschyzon merolae* (Cm) and the green picoalga *Pycnococcus provasolii* (Pp) have both plant-like CRYs and plant CRYs (Figure 3).
5. Dualchrome, a new dual-photoreceptor chimera. Recently, a new type of photoreceptor from the marine picoalga Pp was discovered in metagenome data of ocean picoplankton, named dualchrome (DUC1, Figures 2, 3). DUC1 bears a fusion of a phytochrome and a plant CRY (Makita et al., 2021) and was not found in any other alga or other organisms so far.

### Spatiotemporal Expression of Algal Cryptochromes

#### Expression Profiles in Light–Dark Cycles

The expression of algal cryptochromes was so far mainly studied under light–dark cycles (diurnal conditions). In most cases, it is still unknown if their expression is also controlled by the circadian clock. Algal cryptochromes are routinely expressed in a diurnal way whereby the amplitude of the rhythm and the phase of the peak can vary. The green algal Cr pCRY protein (formerly known as *Chlamydomonas* photolyase homolog 1, CPH1) is nearly absent during the day, but accumulates strongly (amplitude increase up to tenfold) during the night (Reisdorph and Small, 2004; Müller et al., 2017). Cr pCRY protein abundance seems to be independent of the circadian clock (Müller et al., 2017). The protein is known to be rapidly degraded by light *via* the proteasome pathway. Transcript levels of Cr pCRY also increase during the night (Kottke et al., 2017), which is also the case for a plant CRY from the chlorophyte *Haematococcus pluvialis* under the tested high light conditions (Hang et al., 2020). These findings along with the negative regulation of Cr pCRY in gametogenesis (see below) suggests a “dark” function of green algal pCRYs (Müller et al., 2017). In contrast to Cr pCRY, the Cr aCRY protein is rather consistently expressed over the light–dark (LD) cycle (chosen cycle of 12h light:12h darkness), being highest from the beginning until the middle of the day and lowest at the beginning of the night (change in amplitude about 2-fold; Beel et al., 2012; Kottke et al., 2017). The third CRY from Cr, Cr CRY-DASH1 reaches its maximal protein abundance at midday and is lowest at the beginning of the night (change in amplitude about 4-fold; Rredhi et al., 2021). Taken together, the acrophases of peak expression vary with all three investigated Cr CRYs, ranging from early day until late night. For some other algal cryptochromes, only transcript abundances have been determined so far. In the kelp *Saccharina japonica* (Sj), the maximum transcript level of a *CRY-DASH* is reached at midday in a LD 12h:12hcycle (Yang et al., 2020). Transcript levels of the marine picoalga Ot *CPF1*, as determined by microarrays, peak during the day, while levels of Ot *CPF2*, belonging to the CRY-DASH family, peak at the beginning of the day (Heijde et al., 2010). Also in Pt, all studied transcript levels of different *CRYs* (studied by qPCR) had rhythmic expression in an LD cycle of 16h:8h with peaks in different acrophases, indicating a possible functional diversification within the diurnal cycle (Oliveri et al., 2014).

#### Localization of Cryptochromes

Depending on the expression and function, the subcellular localization of CRYs may vary. The green algal Cr aCRY plays a role in vegetative cells as well as in pregametes and gametes (Zou et al., 2017). During daytime (LD4 being 4h after light was switched on in a 12h:12h LD cycle), Cr aCRY is localized to a significant extent in the nucleus of vegetative cells, but is mainly distributed over the cell body (most likely in the cytosol) at late night (LD22 being 10h after light was switched off). In sexual cells (pregametes, gametes, and dark inactivated gametes) Cr aCRY was never found in the nucleus, but always distributed over the cell body. The presence of Cr aCRY in the nucleus of vegetative cells at daytime is in congruence with its activity as (6-4) photolyase, repairing UV-B induced damages (Franz et al., 2018). Thus, Cr aCRY localization varies depending on its function. In contrast, the subcellular localization of Pt CPF1 in the nucleus was not differentially regulated by darkness and light (blue or ultraviolet) treatment (Coesel et al., 2009).

For Cr pCRY, experimental data is lacking, but it is predicted to be localized in the nucleus (Kottke et al., 2017).

In the case of the colony-forming alga *Volvox carteri* (Vc) that consists of somatic cells and sexually active gonidia, transcript levels were determined for different photoreceptors in the different cell types. Vc aCRY and Vc pCRY were found to accumulate during final cellular differentiation (Kianianmomeni and Hallmann, 2015). Some Vc photoreceptor genes, including Vc pCRY, are highly expressed in the somatic cells. These data give first insights into the possibility that the different CRYs may also be involved in developmental cycles.

As mentioned above, CRY-DASH proteins like CRY3 from *A. thaliana* have been shown to be localized in organelles; CRY3 is found in chloroplasts and mitochondria (Kleine et al., 2003). Localization studies revealed the presence of CRY-DASH from the dinoflagellate *Karenia brevis* (Kb) only in chloroplasts (Brunelle et al., 2007). The green algal Cr CRY-DASH1 bearing a chloroplast transit peptide was also found solely in the chloroplast but not in mitochondria, using biochemical fractionation (Rredhi et al., 2021). Its localization is thus in close relation with its biological function, as detailed below.

Localization of the novel type dualchrome was analyzed heterologously in tobacco cells in fusion with GFP. It was primarily found in the nucleus and its localization was not changed by light treatment (Makita et al., 2021).

### Biological Functions of Algal Cryptochromes

Although a large repertoire of algal cryptochromes is known (Figure 3), only a few members have been studied in depth regarding their biological function(s). These studies were mostly done in model algal species that can be transformed and where overexpressing lines and knockdown or knockout mutants can be generated. Table 1 summarizes the main functions of these cryptochromes. Like land plant CRYs, algal CRYs are involved in the regulation of several cellular functions.

**Table 1:** Biological functions of algal CRYs, including comparative data from transgenic lines.

Type	(6-4) repair	(CPD) repair	Circadian clock	Sexual cycle	Photosynthesis: Components, apparatus	Other	References
● Cr pCRY			X	X			Forbes-Stovall et al., 2014; Müller et al., 2017: Kamrani et al., 2018
● Pt CryP					X	X^a^	Juhas et al., 2014; König et al., 2017b
● Cr aCRY	X		X	X	X	X	Beel et al., 2012; Zou et al., 2017; Franz et al., 2018
● Pt CPF1	X		X		X	X	Coesel et al., 2009
● Ot CPF1	X		X				Heijde et al., 2010
● Ot CPF2 (CRY-DASH)		X^b^	X				Heijde et al., 2010
● Cr CRY-DASH1					X	X^c^	Rredhi et al., 2021

a *Photoreceptor network;*

b *weak CPD activity;*

c *growth curve;*

*Cr, Chlamydomonas reinhardtii; Ot, Ostreococcus tauri, Pt, Phaeodactylum tricornutum.*

#### Dual Function Cryptochromes: DNA Repair and More

The first evidence for an algal animal-like cryptochrome with repair activity came from a marine diatom. Pt CPF1 was found to be a member of the cryptochrome (6-4) photolyase family. In contrast to other animal and land plant cryptochromes, Pt CPF1 shows (6-4) photoproduct repair activity (Coesel et al., 2009). Using a Pt *CPF1*-overexpressing line, blue-light regulated transcript levels of wild type were compared to that line. The analyses suggest that Pt CPF1 has a regulatory role in controlling the photosynthetic light harvesting complex and photoprotection as well as cell-cycle progression in addition to its photolyase activity (Coesel et al., 2009). Moreover, a clock function was postulated as detailed in the section below. Similarly, Ot CPF1 from the marine picoalga Ot exerts (6-4) photolyase activity and is involved in circadian rhythms (Heijde et al., 2010). Also the freshwater green alga Cr that lives mainly in wet soil (Sasso et al., 2018) has a dual function cryptochrome, Cr aCRY. It repairs (6-4) photoproducts; a knockdown mutant is less resistant to UV-B treatment than wild type (Franz et al., 2018). In addition, aCRY is involved in regulating transcript levels that are controlled by blue, yellow or red light, including those that encode a light harvesting protein (LHCBM6), glutamine synthetase 1, an enzyme of nitrogen metabolism or the circadian clock component C3, a subunit of the RNA-binding protein CHLAMY1 (Beel et al., 2012).

#### Algal CRYs and the Biological Clock Machinery

As indicated above, dual function animal-like cryptochromes are also involved in the circadian clock machinery. For Pt and Ot CPF1, the dual function was shown by positively testing their repressor activities in a heterologous system. The transcription factors CLOCK and BMAL were used for this purpose. They form a dimer and are part of the mammalian clock-controlled feedback loop. The inhibition of the CLOCK-BMAL-mediated transcription by Pt and Ot CPF1 was positively verified in COS cells using an E-box bearing luciferase reporter system (Coesel et al., 2009; Heijde et al., 2010). Moreover, Ot *CPF1* knockdown lines were generated along with a luciferase reporter that was put under the control of a circadian promoter. In several of these lines, period lengthening (of 1 up to 5h) was observed compared to wild type, and the amplitude was dampened (Heijde et al., 2010). In a Cr *aCRY* knockdown line, light-induced induction of the C3 subunit of the circadian RNA-binding protein CHLAMY1 that influences the acrophase was altered (Beel et al., 2012). These experiments suggest that dual function CPF1/aCRYs play an important role within the circadian system.

For the green algal Cr pCRY, an involvement in the circadian clock was also found, using knockdown lines and a representative circadian clock-controlled process named photoaccumulation or phototaxis. Detection of this rhythm is automated (Forbes-Stovall et al., 2014; Müller et al., 2017). Phase response curves (PRCs) with blue-light pulses taken from wild type and a Cr *pCRY* knockdown line revealed the influence of Cr pCRY on PRC behavior. The largest differences in phase resetting of about 10h between wild type and the mutant were observed in early subjective night (Müller et al., 2017). These data suggest a role of pCRY in the entrainment of the circadian clock. Moreover, the Cr *pCRY* knockdown line exerts a significantly lengthened period of about 27.9h compared to wild type (24.5h in average); it becomes arrhythmic after a few days under free-running conditions that are used to analyze clock properties. It was also suggested that pCRY positively regulates the clock component, ROC75, a putative transcription factor (Kamrani et al., 2018). These data support the model that Cr pCRY is not only involved in circadian input but is also linked to the central oscillator.

#### CRYs and Life Cycle Regulation

The sexual cycle of the unicellular alga Cr, which is regulated by light and nitrogen availability [reviewed in Kottke et al. (2017); Sasso et al. (2018)], is well studied. Cr vegetative cells of both mating types (mt^+^ and mt^−^) turn into pregametes in the dark upon the removal of the nitrogen source. In the presence of light and without nitrogen source, pregametes become gametes. This process is controlled by three photoreceptors. It is promoted by phototropin and inhibited by two algal cryptochromes, aCRY and pCRY (Huang and Beck, 2003; Müller et al., 2017; Zou et al., 2017). It is assumed that the inhibition process allows gametes to reconvert to pregametes and vegetative cells if a nitrogen source becomes available short term. Sexually active gametes finally fuse to form a quadriflagellated cell that is converted to a resilient zygote (Figure 1). Zygotes can survive in the dark and without a nitrogen source for months (Sasso et al., 2018). Upon light and nitrogen availability, germination is induced, and a tetrad of haploid vegetative cells is formed which results in individual vegetative cells. Activation of germination is again mediated by the above-mentioned three photoreceptors (phototropin, aCRY, and pCRY), but in this case all three act in concert as positive regulators (Huang and Beck, 2003; Müller et al., 2017; Zou et al., 2017). Thus, both aCRY and pCRY play central roles in the sexual cycle of Cr either as negative regulators (gamete formation) or as positive regulators (germination). In this context, it is also of interest that blue light is the main trigger for gamete formation and germination. However, red light also has a significant albeit smaller influence on germination (Zou et al., 2017). aCRY with its property to absorb also in the red spectral region in addition to blue (Beel et al., 2012, see also chapter on properties below) seems to be the relevant receptor for red light because pCRY and phototropin do not absorb in this range.

#### CRYs and the Photosynthetic Apparatus Regulation

CRYs have a broad influence on the photosynthetic machinery by regulating transcript levels and/or protein abundance of photosynthetic components, pigments or the entire photosynthetic machinery. Here, we will present exemplarily the effects of two representative CRYs, the plant-like CryP from Pt and CRY-DASH1 from Cr, but it should be mentioned that also Pt CPF1 and Cr aCRY were shown to be involved in the regulation of transcript levels of photosynthetic compounds (Coesel et al., 2009; Beel et al., 2012).

Plastids and the photosynthetic apparatus of diatoms are different from that of Chloroplastida in several aspects. Diatoms belong to the secondary red endosymbionts (Figure 3). Their plastids are surrounded by four membranes, and they possess additional fucoxanthin pigments that give them an orange, brownish color. Based on a Pt *CryP* knockdown line, effects of this plant-like CRY on proteins of the photosynthetic apparatus were studied. Here, the most prominent members of the diatom light harvesting chlorophyll-fucoxanthin family (Lhcf) as well as proteins involved in their photoprotection (Lhcx) were analyzed. Lhcf1 -Lhcf11 protein abundance was enhanced in the *CryP* knockdown strains in comparison to wild type cells (Juhas et al., 2014). Contrarily, the protein level of Lhcx was decreased in the knockdown lines (Juhas et al., 2014). Changes were also observed in transcript levels under slightly different conditions, but were not always in agreement with the protein levels suggesting that posttranscriptional regulation plays a role (König et al., 2017a). Taken together, these data show that CryP contributes significantly to changes in photosynthetically relevant compounds.

Characterization of the green algal Cr CRY-DASH1 protein represents a first functional in-depth study of an algal CRY-DASH protein. The Cr *CRY-DASH1* knockout line showed a significantly reduced growth rate (Rredhi et al., 2021) in contrast to Pt *CryP* knockdown lines that were influenced in photosynthesis components, but had a similar growth rate to wild type (Juhas et al., 2014). Intriguingly, the content of chlorophyll a and b as well as of the carotenoids was increased in the *CRY-DASH1* knockout line. This was even visible by the naked eye; cultures of the knockout line were of a darker green than wild type (Rredhi et al., 2021). The increase in pigments went hand in hand with hyper-stacking of thylakoid membranes and an increase in two of the central proteins of photosystem II, D1 and the antenna protein CP43 in the mutant line (Rredhi et al., 2021). CRY-DASH1 thus acts as a repressor that prevents the synthesis of excessive pigments and membranes and thus balances the photosynthetic machinery. Its regulatory role seems to be exerted at the posttranscriptional/translational level as the transcript levels of the genes encoding D1 and CP43 are not altered in the mutant compared to wild type (Rredhi et al., 2021). It is postulated that the observed reduction in growth is due to the higher pigment amount resulting in a shading effect. Indeed, light intensity within a culture flask of the knockout line was reduced compared to that of a wild-type-containing-flask (Rredhi et al., 2021). Notably, Cr CRY-DASH1 absorbs primarily in the UV-A range (see chapter below), where the photosynthetic pigments absorb only to a smaller extent.

#### Others

The recently described dualchrome (DUC1) represents a chimera consisting of a phytochrome and a cryptochrome in the rather unknown green picoalga Pp (Figure 2). It was found that the Pp genome encodes also further homologue proteins of the model plant *A. thaliana* involved in light signaling such as phototropin, CONSTITUTIVE PHOTOMORPHOGENIC 1 (COP1), or ELONGATED HYPOCOTYL 5 (HY5) in addition to the mentioned CRY variants (Figure 3). Thus, it possesses a gene set necessary for adapting to various light conditions (Makita et al., 2021).

The presence of several CRYs and other photoreceptors in algae elicits the question of if they form a photoreceptor network influencing each other. Indeed, it was found in Pt *CryP* knockdown lines that the transcript levels of other photoreceptors (Pt phytochrome and Pt CPF1) are influenced by the reduction of Pt CryP. These data suggest exactly such a scenario (König et al., 2017a).

### Biophysical Properties of Algal Cryptochromes

The physiological responses to light are the result of a chain of events on the molecular level. First, light is absorbed by a chromophore bound to the receptor, which then leads to a photochemical reaction. This reaction causes a change in structure and/or conformation of the protein, which then may change the interaction of the receptor with a signaling partner such as a specific binding protein or DNA. Finally, a signal transduction cascade leads to a change in the physiology of cells and organisms.

#### Light-Induced Oligomerization of Cryptochromes

An interesting aspect of the cryptochrome response to light is that the photochemical reaction of the FAD may cause a change in the oligomerization state, i.e., in the number of identical receptors that associate within a dynamic complex. A formation of large complexes called photobodies of At CRY2 has been observed in plant cells (Mas et al., 2000), which is in agreement with a homooligomerization to clusters of receptors upon blue-light illumination (Bugaj et al., 2013). These clusters are reversible in the dark and dissociate within minutes. Accordingly, the reversible clustering has drawn much attention to the development of optogenetic tools to localize fusion proteins to one spot within mammalian cells by illumination (Bugaj et al., 2013; Taslimi et al., 2014). Recent structural characterization by cryo-electron tomography and X-ray crystallography has revealed that At CRY2 forms tetramers of the PHR upon illumination (Ma et al., 2020; Shao et al., 2020; Palayam et al., 2021).

Oligomerization of algal cryptochromes has been studied on some selected members and light-induced changes in oligomerization have been found. A similar clustering behavior to At CRY2 has been observed for the light-sensitive domain of algal Cr pCRY as a fusion protein labeled with a fluorescence protein. The light-induced degradation of Cr pCRY in *C. reinhardtii* prevents a study of a potential functional role of this clustering *in vivo* (Reisdorph and Small, 2004; Müller et al., 2017). However, it has been shown that a complex containing Cr pCRY is formed at the end of the night in *C. reinhardtii*, possibly in context with its dark-related function. It remains open whether this complex includes homooligomers of pCRY or if it is a heteromeric complex with yet unknown partner(s) (Müller et al., 2017).

The oligomerization state has been characterized for isolated Cr aCRY in great detail. Here, red light induces the transition from a dimer in the dark to a tetramer in the light (Oldemeyer et al., 2016). Interestingly, the presence of the CCT is required for the formation of the dimer in the dark. Truncation of the CCT produces a monomer, which forms a dimer upon illumination only to a small extent (Oldemeyer et al., 2016). Some deviating results on oligomerization states of Cr aCRY can be explained by the different exclusion volumes of the columns used for gel filtration (Franz-Badur et al., 2019).

In summary, light-induced changes in oligomerization state have been observed in algal cryptochromes but differ between pCRY and aCRY. In general, little is known about complex formation of algal cryptochromes with signaling partners or other photoreceptors. These signaling partners need to be identified to link the changes in oligomerization state to the signal transduction cascade.

#### Strong Variations in the Absorption Spectra Define the Different Roles of Algal Cryptochromes

The absorption spectrum of a photoreceptor in the dark is decisive for the region of the sun’s spectrum, by which this receptor is activated. Ideally, the absorption spectrum matches the action spectrum that was recorded for the function *in vivo*. The absorption spectrum of cryptochromes reflects the redox state of the FAD cofactor, i.e., whether it binds oxidized FAD (FAD_ox_), the anion radical (FAD•^−^), the neutral radical (FADH•), or fully reduced FAD (FADH^−^). Different redox states of FAD can be stabilized in the dark by the specific protein environment of a cryptochrome. Accordingly, a cryptochrome acts as a blue and UV-A light receptor, if (FAD_ox_) is the stable redox state in the dark.

The interpretation of the absorption spectra of cryptochromes is complicated by the fact that usually the recording is performed after isolation from a heterologous expression system, which might lead to an oxidation of the FAD. In addition, an antenna chromophore is bound to some cryptochromes, which might be lost upon purification or might not even be produced by the heterologous expression system. The antenna molecule, which has a higher extinction coefficient as compared to flavin, serves the purpose to absorb the light and to transfer energy to the FAD cofactor, thereby increasing the probability of light absorption.

**Figure 4:**
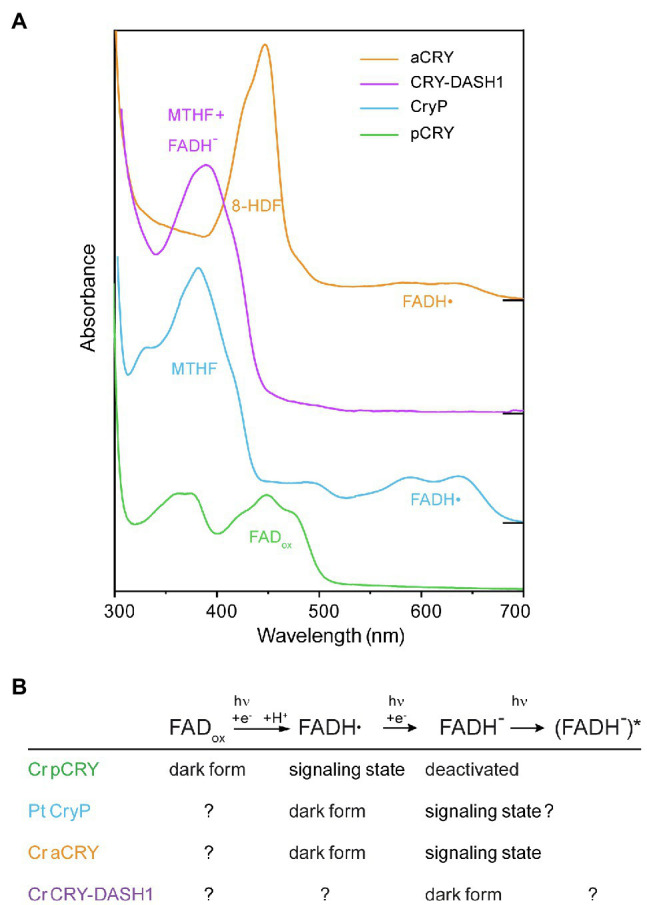
Absorption spectra of different cryptochromes from algae recorded *in vitro* directly after isolation and the postulated roles of the redox states of FAD in these cryptochromes. **(A)** The absorption spectra of cryptochromes are characterized by the different oxidation states of the FAD chromophore and the binding of antenna molecules. The plant cryptochrome Cr pCRY binds FAD_ox_ and acts as blue-light receptor. Plant-like cryptochrome Pt CryP and animal-like cryptochrome Cr aCRY both absorb almost in the full visible spectral region because of the absorption of FADH•. However, Pt CryP binds MTHF as antenna, whereas Cr aCRY binds 8-HDF leading to different absorption maxima in the UV-A and blue region, respectively. The UV-A-receptor Cr CRY-DASH1 absorbs mainly at around 380nm dominated by the contribution of MTHF, whereas its chromophore FADH^−^ absorbs only weakly. For simplicity, the absorption of FAD_ox_, FADH• and FADH^−^ is indicated at the maximum with highest wavelength, but all FAD species also contribute to the absorption at lower wavelengths. Spectra were displaced vertically for better visibility. **(B)** The redox states of FAD play different roles in the algal cryptochromes, as postulated on the basis of the absorption spectra and functional studies of representative members. The dark form indicates the redox state present *in vivo* in the dark. Light absorption induces a reduction of the FAD. The signaling state is associated with conformational changes and/or changes in oligomerization state of the receptor that drive signal transduction.

The plant cryptochrome Cr pCRY shows a typical spectrum for a blue-light receptor carrying oxidized FAD (FAD_ox_) with maxima in the blue and UV-A region (Figure 4A; Immeln et al., 2007). The presence of the CCT does not influence the absorption spectrum (Goett-Zink et al., 2021). Some residual MTHF has been identified after purification from bacterial cells, but a functional role as an antenna chromophore and binding of MTHF in the alga is not likely. FAD_ox_ in pCRY is even present in the strongly reducing cytosol of living *Escherichia coli* cells (Goett-Zink et al., 2021), which is a strong argument for FAD_ox_ being the native chromophore of pCRY in *Chlamydomonas* in the dark similar to findings for pCRYs from land plants (Bouly et al., 2007).

Members of the animal-like CRY/CPF1 family such as Ot CPF1, Pt CPF1 and Cr aCRY bind FAD_ox_ after purification (Coesel et al., 2009; Heijde et al., 2010; Beel et al., 2012). The more unusual was the finding that Cr aCRY uses the flavin neutral radical (FADH•) as the dark state for a sensory function thereby accessing almost the complete visible spectral region including yellow and red light (Beel et al., 2012; Figure 4A). This fact suggests that FADH• is the predominant form of FAD in the alga in the dark, which is supported by a very efficient photoactivation reaction from FAD_ox_ to FADH• *in vitro* (Lacombat et al., 2019). The tight binding of an antenna molecule is another difference of aCRY/CPF1 to plant cryptochromes. The binding of 8-HDF was suggested for Ot CPF1 from structural considerations (Brazard et al., 2012) and then identified for Cr aCRY after implementing the synthesis of 8-HDF in *E. coli* (Franz et al., 2018). The antenna 8-HDF strongly absorbs light in the blue region of the visible spectrum (Figure 4A) and additionally stabilizes the FADH• in the dark, preventing oxidation (Oldemeyer et al., 2020).

Much less is known about the plant-like subfamily and its member Pt CryP. The binding of stable FADH• and MTHF in the dark after purification might indicate a light response similar to the aCRY family, with the distinct difference that the antenna MTHF absorbs shifted to the UV-A region compared to 8-HDF (Figure 4A; Juhas et al., 2014). Functional studies have focused so far only on the blue-light effects of CryP on Pt physiology (König et al., 2017a). It would be of high interest to study also potential responses of Pt CryP to green and red light.

The large subfamily of CRY-DASH proteins (Brudler et al., 2003) is also well represented with several members in algal genomes (Figure 3). Few members have been isolated and characterized by absorption spectroscopy. A typical behavior for members of CRY-DASH has been found for Ot CPF2 *in vitro*, which showed a dominant absorbance by the antenna MTHF and some contribution from FAD_ox_ (Heijde et al., 2010). Similar findings have been presented for Cm PHR5 (Asimgil and Kavakli, 2012). Recent investigations on Cr CRY-DASH1 revealed that FAD is present in the fully reduced state (FADH^−^) directly after purification, which together with the photochemical results indicates a presence of FADH^−^ in Cr *in vivo* (Rredhi et al., 2021). As a result, Cr CRY-DASH1 mainly absorbs light in the UV-A-region (Figure 4A), supported by functional studies as a UV-A receptor in Cr. Of note, the UV-A absorption of CRY-DASH1 is maximal in a spectral region where the typical blue-light receptors pCRY and phototropin as well as photosystems I and II show only weak absorption. Accordingly, CRY-DASH1 may complement the other receptors in the alga by the spectral range in which it is activated.

In summary, the strong differences in the absorption spectra of cryptochromes in the dark reflect the variety of functions for which these receptor subfamilies evolved (Kottke et al., 2017; Figure 4B). Cryptochromes act in algae as UV-A receptor (Cr CRY-DASH1), as UV-A/blue-light receptor (Cr pCRY) and as “white light” receptor covering almost the full spectral region (Cr aCRY), defined by the redox state of FAD cofactor in the dark. The FAD is reduced after the absorption of light and forms the signaling state, accompanied by conformational changes and/or changes in oligomerization state of the receptor. The identification of the signaling state and the mechanism of signal propagation within the receptor are important biophysical questions to be answered for each member. The details of the different pathways identified to date go beyond the scope of this review. Even more differences in the light responses will be revealed as more and more candidates of algal cryptochromes are being characterized biophysically.

## Outlook

It is expected that metagenome analysis from phytoplankton will lead to the discovery of even more novel types of photoreceptors, including new cryptochrome variants such as the recently identified DUC1. The high number of different light sensors in aquatic organisms is correlated with the fact that light quality and light quantity for aquatic algae change dynamically. The light conditions depend on the depth within the water column where the algae occur at a given time in the ocean, on the presence of solved particles and on other light-absorbing organisms at this location. The challenge will be to analyze the role of all these photoreceptors and their mutual interaction partners in the *in vivo* systems.

Algae represent a rich source for novel cryptochrome photoreceptors. These might be used as optogenetic tools to generate light switches in algal cells for controlling gene expression or in combination with signaling molecules such as cAMP or Ca^2+^ (Kianianmomeni and Hallmann, 2014). They might trigger light-dependent adaptations of organismal physiology, development, and behavior in nature (Losi et al., 2018) and thus create potent tools for biotechnological approaches. They may also be used in heterologous systems for neurobiology and in medicine, as it was recently done with an algal rhodopsin to partly recover vision in blind people (Sahel et al., 2021). The land plant At CRY2 was already used for optogenetical protein clustering (Park et al., 2017) and for regulating gene expression in mammalian cells (Hernández-Candia et al., 2019). In general, the high variety of algal CRYs offers a high potential for engineering new sensor tools.

Currently, we are only beginning to understand the biophysical properties and the involved signal transduction pathways as well as the biological functions of the different algal cryptochromes. Their interplay within one algal system and along with the other photoreceptors of this system remains largely enigmatic, even in the today selected model algal organisms. Double-, triple-, or even higher-order mutants need to be produced to gain further insights in their complex photobiology. Yet, the basis of these light signaling events is the key to understand (i) algal fitness and thus (ii) their central contribution as photosynthetic organisms to life on Earth. As our knowledge on the role and function of algal cryptochromes improves, it will be possible to manipulate growth, reproduction and the development of algae as well as to optimize photosynthesis, for example by application of targeted illumination protocols.

## Author Contributions

JP, AR, JS, and MM designed and drew Figures 1–3. SO and TK created Figure 4. JP and MM arranged Table 1. JP, AR, TK, and MM wrote the paper, with input from all authors. All authors contributed to the article and approved the submitted version.

## Funding

Our work was supported by the Deutsche Forschungsgemeinschaft by DFG grants Mi373/16-1 to MM and Ko3580/4-2 and Ko3580/7-1 to TK. SO was funded by the DFG Sonderforschungsbereich SFB1078.

## Conflict of Interest

The authors declare that the research was conducted in the absence of any commercial or financial relationships that could be construed as a potential conflict of interest.

## Publisher’s Note

All claims expressed in this article are solely those of the authors and do not necessarily represent those of their affiliated organizations, or those of the publisher, the editors and the reviewers. Any product that may be evaluated in this article, or claim that may be made by its manufacturer, is not guaranteed or endorsed by the publisher.
